# Impact of continuous *Panax notoginseng* plantation on soil microbial and biochemical properties

**DOI:** 10.1038/s41598-019-49625-9

**Published:** 2019-09-13

**Authors:** Yu Zhang, Yujie Zheng, Pengguo Xia, Lulu Xun, Zongsuo Liang

**Affiliations:** 1 Laboratory of Plant Secondary Metabolism and Regulation of Zhejiang Province, College of Life Sciences and Medicine, Zhejiang Sci-Tech University, Hangzhou, 310018 China; 2 Institute of Soil and Water Conservation, Chinese Academy of Sciences & Ministry of Water Resources, Yangling, 712100 China; 3 State Key Laboratory of Membrane Biology, Innovation Center for Structural Biology, School of Life Sciences, Tsinghua University, Beijing, 100084 China

**Keywords:** Plant ecology, Soil microbiology

## Abstract

*Panax notoginseng* is a highly regarded medicinal plant that has been cultivated for more than 400 years in Southwest China. The obstacles associated with the continuous cropping of *P. notoginseng* are the greatest issues for the development this plant. In the present study, the micro-ecologies of soils differing in the duration of *P. notoginseng* planting were compared, the results of which could provide important information to aid in solving the problems associated with the continuous cropping of *P. notoginseng*. Soils in which *P. notoginseng* had grown for 1, 3 or 5 years, as well as unplanted or fallow soil, which had a *P. notoginseng* planting interval of 1, 3, 6 or 9 years, were collected in Yunnan Province, China. The numbers and physiological groups of microorganisms, soil enzyme activities and nutrients present in the soil were analyzed to identify the effects of continuous cropping and determine the influence of crop rotation on the soil. After *P. notoginseng* was planted, the ecological structure of the soil and the balance of soil nutrients changed. These changes in the soil ecosystem prevented the soil from adapting to the continuous cropping of *P. notoginseng*, which eventually limited the growth of *P. notoginseng* and increased the incidence of diseases. After rotation of *P. notoginseng*, some soil indicators were restored, and some indicators with irregular changes may have been caused by crop rotation and field fertilization management practices. Thus, the selection of suitable crop rotations will facilitate the use of continuous cropping for *P. notoginseng*.

## Introduction

*Panax notoginseng* (Burk.) F.H. Chen is a highly regarded medicinal plant that has been cultivated for more than 400 years in Southwest China, particularly in Yunnan Province. This plant has many important properties, such as antihypertensive, antithrombotic, anti-atherosclerotic and neuroprotective activities^1^ and is used for the treatment of cardiovascular diseases, inflammation, various body pains, traumas, and internal and external bleeding due to injury^2^. Continuous cropping, in which the same crop or related crops are continuously planted, decreases the yield and quality of crops^3,4^. These obstacles associated with continuous cropping have become a major constraint in *P. notoginseng* cultivation as the land suitable for planting *P. notoginseng* is limited. Such obstacles have led to an increase in the costs of planting *P. notoginseng* and decreases in the yield and quality of *P. notoginseng*^5,6^. Generally, it should wait many years for *P. notoginseng* to be planted for a second time after it has been harvested^7^.

The primary issues associated with continuous cropping include changes in soil microbes, physicochemical properties and allelopathy^8–11^. Although there are many issues associated with continuous cropping, the most fundamental ones include promoting an imbalance in soil microbiota and diversity, a reduction in beneficial microorganisms, and an enrichment of pathogenic microorganisms and various soil-borne plant diseases^12^. More importantly, interactions among the various factors associated with to continuous cropping further exacerbate the occurrence of these obstacles^13,14^. Continuous cropping is closely affected by the types, numbers, and diversity of soil microorganisms^14,15^. Changes in soil microbial biomass, diversity, and microbiota directly affect crop growth. Previous studies have demonstrated that changes in soil microbiota are the major causes of the obstacles associated with the continuous cropping of *P. notoginseng*^4–7,16,17^.

Due to the limited growing area of *P. notoginseng*, it is imperative to conduct research on the effects of continuous cropping. We hypothesized that soil nutrients, soil microorganisms and soil enzymes would change in response to continuous cropping. Our previous research focused on assessing the yields of volatile oils, the level of saponins, the utilization of water and fertilizer, and the effects of a three-year growth pattern of *P. notoginseng*; however, the soil microbial and biochemical properties of this system have not been studied^18,19^. Therefore, in this study, soils currently and previously planted with *P. notoginseng* were used to study the changes in soil microbes, enzymes, and nutrients during the process of planting *P. notoginseng*. These changes revealed possible modifications to the soil cause by continuous cropping, providing detailed information on the continuous cropping method used to grow *P. notoginseng*.

## Materials and Methods

### Collection of soil samples

The experimental soil samples were collected from Wenshan Autonomous Prefecture, Yunnan Province (103°35~104°45E, 23°18~23°59N). The altitude of the study area is 1539 m, with an average annual temperature of 16.1 °C and annual rainfall of 1008 mm. In addition, the area has 250 to 320 frost-free days and is located within the primary *P. notoginseng* producing areas. Soils in which *P. notoginseng* had been grown for 1, 3 or 5 years and those from fields previously planted with *P. notoginseng* but allowed to fallow for 1, 3, 6 or 9 years were collected. The soils were collected from seven sites in Wenshan and the sampling strategy included collecting soil around *P. notoginseng* plants, with multipoint sampling also used. The soils for every site were collected three times. All soil samples were placed in sterile bags, and each sample was divided into two subsamples: one portion was air-dried for soil enzyme activity and nutrient analysis, and the others was stored at 4 °C for microbiological experiments^20^.

### Analysis of different species of soil microorganisms

Ten grams of soil was placed into 90 ml of sterile water and shaken well, after which the mixture was diluted in a gradient to different final concentrations. The numbers of bacteria, fungi, and actinomycetes were determined by the serial dilution plate count method. Serial dilutions of 10^−4^–10^−6^ for bacteria, 10^−1^–10^−3^ for fungi, and 10^−3^–10^−5^ for actinomycetes were prepared. Dilutions of different samples were prepared with three replicates, with 0.2 ml of suspension from each serial dilution spread over selective agar medium (Duchefa Biochemie, Holland) for isolation with the medium containing beef extract peptone, rose bengal medium, and Gao I medium. The bacteria, fungi, and actinomycetes were incubated at 28 °C for 2 days, 4 days, and 7 days before counting, respectively^21^.

### Analysis of microbial physiological groups

The study of soil microorganism physiological groups was primarily performed based on the Manual of Soil Microorganism Analysis^22^ and Soil Microorganism Research Methods^23^. The differences in microorganisms were measured by the dilution or plate method. The most-probable-number (MPN) procedure was used to count the microorganisms and each sample was counted three times.

### Identification of soil fungi

The fungi were identified based on the literature^24,25^ and the morphological characteristics of fungal colonies grown in PDA medium were observed. Using PDA medium insertion and patches, the mycelial and spore morphologies were observed with a microscope to identify the fungal species.

### Analysis of soil enzyme activity

Soil enzyme activities were assayed by the buffer method^26,27^. First, invertase, amylase and cellulase activity was measured by the salicylate colorimetric method. Phosphatase activity was measured by the colorimetric method using disodium phenyl phosphate. Catalase activity was measured by the titrimetric method using potassium permanganate. Urease activity was measured by the colorimetric method and polyphenol oxidase and peroxidase activities were measured by the colorimetric method using pyrogallic acid^28^. Enzyme activity was expressed in the following units: invertase, amylase, phosphatase and urease: mg/(g·24 h); cellulase: mg/(10 g·72 h); catalase: ml of 0.1N·KMnO_4_/(g·20 min); and polyphenol oxidase and peroxidase: mg/(g·2 h). All samples were assayed three times.

### Analysis of soil nutrients and organic matter

Soil organic matter was determined using the potassium dichromate volumetric method; available nitrogen was analyzed using an AA3 continuous flow analyzer (SEAL, Germany); available phosphorus was measured by the Mo-Sb colorimetric method; available potassium was determined with the flame photometric method; the water-soluble calcium and magnesium contents were measured by titration using EDTA; and the microelements of soil were determined using the atomic absorption method^29–31^. All samples were tested three times.

### Data analysis

All experimental data were managed using Microsoft Excel 2016. The data were analyzed by one-way ANOVA and Duncan’s multiple comparison test using IBM SPSS Statistics 16.0. Statistical significance was considered at *p* < 0.05.

## Results

### Numbers of soil microbes in different cultivated soil

The results showed that the total numbers of bacteria, actinomycetes and microorganisms differed significantly among different cultivation durations, while the numbers of fungi did not show significant differences (Table 1). The numbers of bacteria and actinomycetes in fallow soil were higher than that in soil with different planting durations and first decreased and then increased with years since cultivation. The numbers of fungi in soil with *P. notoginseng* was lower than that observed in fallow soil.

**Table 1:** Numbers of microorganisms in different cultivated soil.

Planting Year	Bacteria (10^8^ cfu/g)	Fungi (10^5^ cfu/g)	Actinomyces (10^6^ cfu/g)	Total (10^8^ cfu/g)
Fallow fields	5.065a	4.122a	7.070a	5.139a
1 year	3.845b	4.061a	5.616b	3.905b
3 years	3.397b	3.882a	3.971c	3.441b
5 years	4.006b	3.885a	6.070b	4.071b

Notes: Different lowercase letters indicate significant differences (n = 9, *p* < 0.05).

### Numbers of physiological groups in different cultivated soils

**Figure 1:**
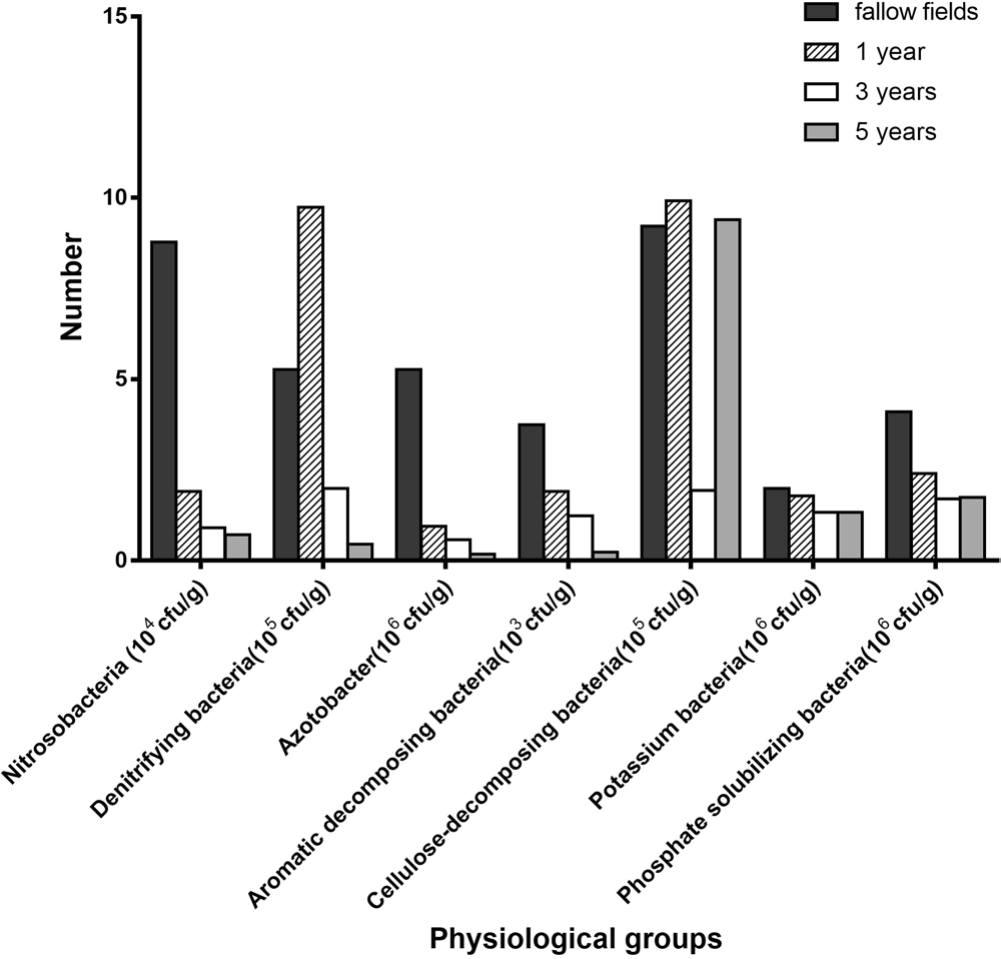
Numbers of physiological groups in different cultivated soils (fallow fields and soils growing *P. notoginseng* for 1, 3 or 5 years).

Analysis of physiological groups revealed differences between fallow soiled and planted soil (Fig. 1). The numbers of nitrosobacteria, aromatic- decomposition microbes, *Azotobacter*, potassium-solubilizing bacteria and phospho-bacteria detected in cultivated soil were lower than those observed in fallow soil. Nitrosobacteria were 4.6–12.2 times more abundant in fallow soil than in the cultivated soil. The numbers of denitrifying bacteria first increased and then decreased with years of cultivation. The numbers of *Azotobacter* and aromatic-decomposing microbes in the soil decreased with years of cultivation. The numbers of cellulose-decomposing bacteria detected in soil with *P. notoginseng* for 3 years was much lower than that in fallow and other cultivated soils. Planting of *P. notoginseng* did not affect the microbial community in the soil, especially with respect to nitrosobacteria, aromatic-decomposing microbes, *Azotobacter* and phosphate-solubilizing bacteria.

### Fungi in different cultivated soils

Members of the genera Rhizopus, Mucor, Botrytis, Saccharomyces, Paecilomyces, Aspergillus, and Mortierella were all detected in soils with different planting durations. Trichoderma was only isolated from fallow fields and from soil in which *P. notoginseng* had been grown for 1 year. Fusarium was only in detected soil with *P. notoginseng* (Table 2).

**Table 2:** The fungal taxa detected in different cultivated soils.

Species	Fallow fields	1 year	3 years	5 years
Rhizopus	√	√	√	√
Mucor	√	√	√	√
Trichoderma	√	√	○	○
Botrytis	√	√	√	√
Saccharomyces	√	√	√	√
Penicillium	√	√	√	√
Paecilomyces	√	√	√	√
Aspergillus	√	√	√	√
Mortierella coemans	√	√	√	√
Fusarium	○	√	√	√

### Enzyme activity in different cultivated soils

The activities of invertase, amylase, catalase and polyphenol oxidase in fallow soil were higher than those in cultivated soil, but that of cellulase first decreased and then increased with years of cultivation. The urease activity, which was the highest in soil in which *P. notoginseng* had been growing for 1 year, showed no regular pattern. Phosphatase activity primarily attributable to acid phosphatase, alkaline phosphatase and neutral phosphatase. Table 3 shows that the phosphatase activity trend in the soils with respect to Ph was acidic >neutral >alkaline. The peroxidase activity in soil in which *P. notoginseng* had been grown for 3 years was much higher than that detected in fallow soil. The activity of polyphenol oxidase first decreased, then increased, and then decreased with years of cultivation. However, the activity of polyphenol oxidase in soil with *P. notoginseng* was significantly lower than that detected in fallow soil (Table 3).

**Table 3:** The enzyme activities detected in different cultivated soils.

Planting Year	Invertase (mg/(g·24 h))	Amylase (mg/(g·24 h))	Cellulase (mg/(10 g·72 h))	Catalase (0.1 N·KMnO_4_ ml)	Urease (mg/(g·24 h))
unplanted	362a	8.72a	36.6ab	1.08a	1.516c
1 year	263b	8.30a	31.9b	0.855b	2.494a
3 years	64.7c	3.51b	22.3c	0.382c	1.080d
5 years	68.3c	1.66c	41.4a	0.773b	1.935b
**Planting Year**	**Phosphatase (mg/(g·24 h))**			**Peroxidase (mg/(g·2 h))**	**Polyphenol Oxidase (mg/(g·2 h))**
	**acidic**	**neutral**	**alkaline**		
unplanted	294a	200a	196a	3.85b	2.54a
1 year	307a	119b	82.3b	4.01b	1.02b
3 years	183b	38.6c	26.2d	9.93a	1.81c
5 years	273a	123b	63.8c	4.53b	1.23b

Notes: Different lowercase letters indicate significant differences (n = 9, *p* < 0.05).

### Soil nutrients in different cultivated soils

**Figure 2:**
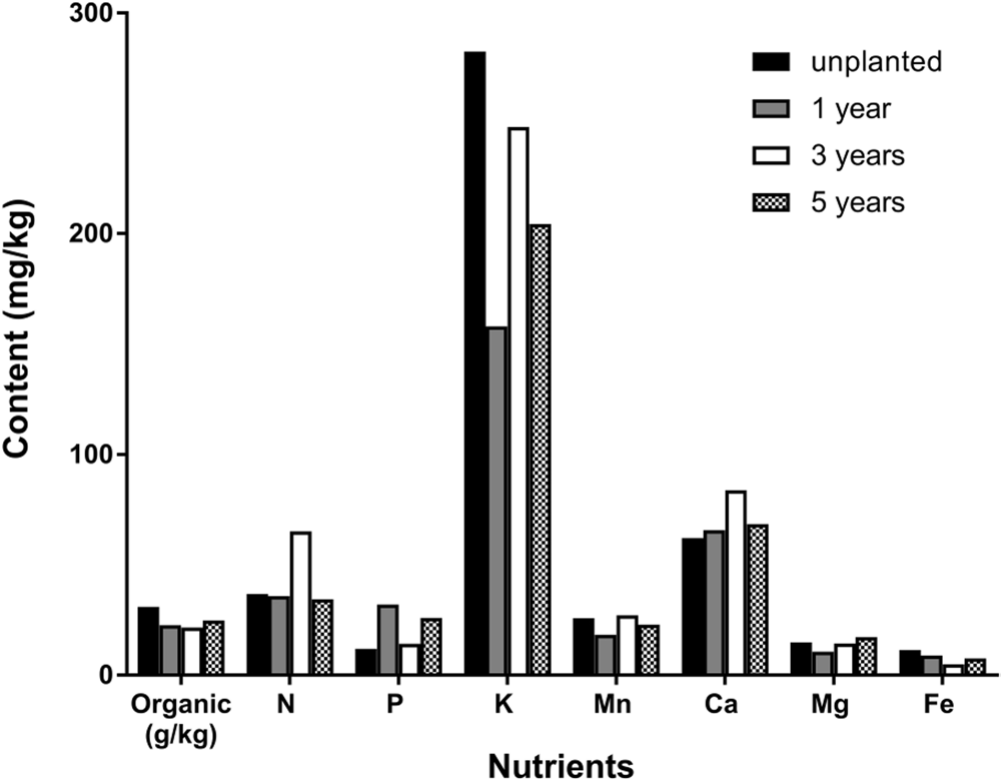
Analysis of soil nutrients (N, P, K, Mn, Ca, Mg, and Fe) in different cultivated soils (fallow fields and soils growing *P. notoginseng* for 1, 3 or 5 years).

**Figure 3:**
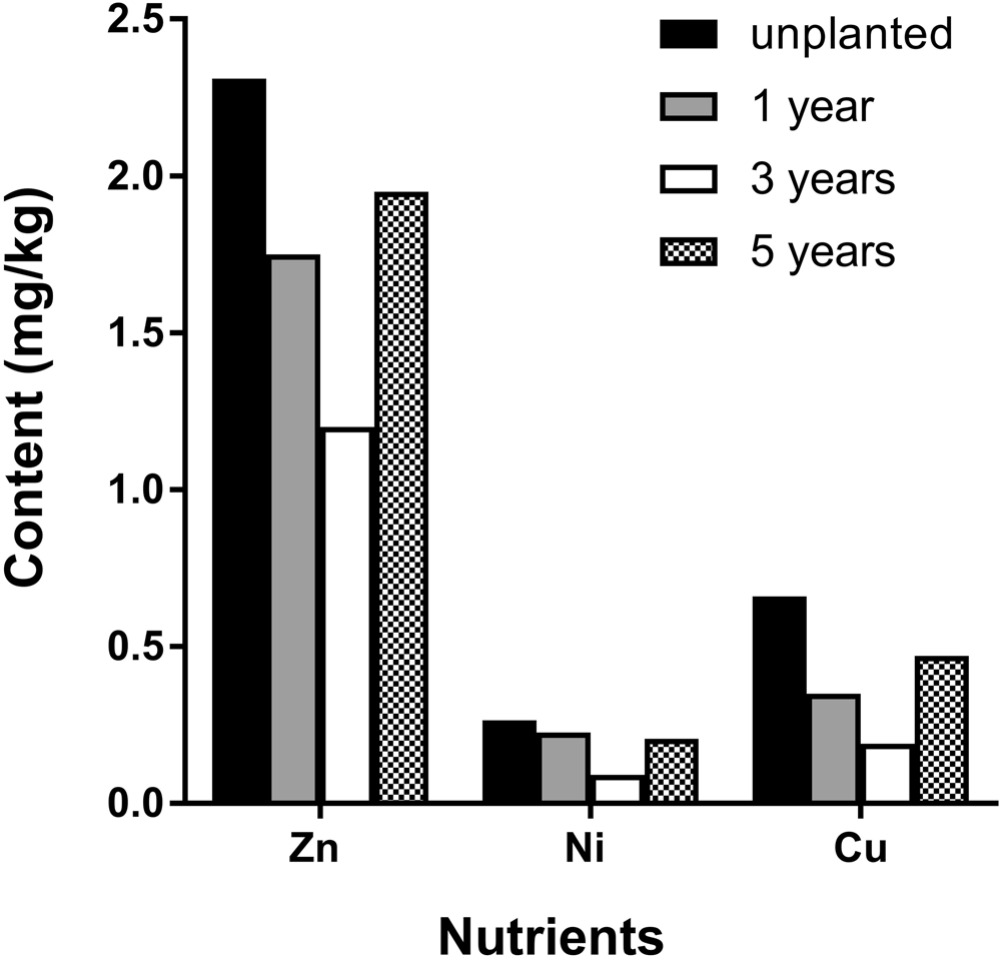
Analysis of soil nutrients (Zn, Ni, and Cu) in different cultivated soils (fallow fields and soils growing *P. notoginseng* for 1, 3 or 5 years).

The organic matter content first decreased and then increased with planting duration. The organic matter in soil with *P. notoginseng* was lower than that detected in fallow soil. The change in available N was irregular, and the available N content was highest in soil in which *P. notoginseng* had been grown for 3 years. Changes in available P, available Mn and water-soluble Mg showed no regular pattern. The available P content in soil in which *P. notoginseng* had been grown for 1 or 5 years was higher than that detected in soil in which *P. notoginseng* had been grown for 3 years and in fallow soil. The available K content in soil with *P. notoginseng* was lower than that detected in fallow soil. With an increase in years of planting, the available K content first decreased, then increased, and then decreased. The available Ni, Cu, Zn and Fe contents in soil with *P. notoginseng* were lower than those detected in fallow soil, showing a trend of first decreasing and then increasing. In contrast, the water-soluble Ca was higher in soil with *P. notoginseng* than in fallow soil (Figs 2 and 3).

### Numbers of soil microbes after different planting intervals

There were significant differences in the numbers of microbes in soils with different planting intervals (Table 4). Except for the interval of 1 year, the numbers of fungi did not show significant differences. The numbers of bacteria in the soil with an interval of 9 years were the lowest and increased with shorter planting intervals. However, the opposite trend observed for actinomycetes, the abundance of which decreased with shorter planting intervals, which was the same pattern observed for the total number of microbes. We also observed that the numbers of microbes in unplanted soil and after an interval of 3 years were similar.

**Table 4:** Numbers of microorganisms present after different interval year.

Interval Year	Bacteria (10^8^ cfu/g)	Fungi (10^5^ cfu/g)	Actinomyces (10^6^ cfu/g)	Total (10^8^ cfu/g)
fallow fields	3.910b	2.862a	0.726c	3.920b
9 years	1.012d	3.117ab	3.198a	1.047d
6 years	2.648c	2.994ab	1.267b	2.654c
3 years	4.022b	3.218ab	0.613cd	4.031b
1 year	5.597a	3.731b	0.498d	5.605a

Notes: Different lowercase letters indicate significant differences (n = 9, *p* < 0.05).

### Numbers of physiological groups after different planting intervals

**Figure 4:**
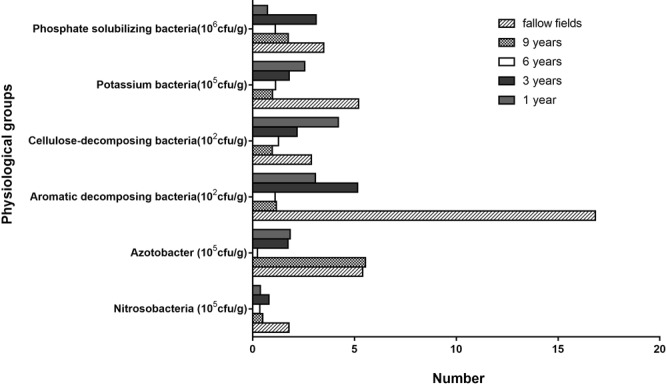
Numbers of physiological groups after different interval years (1, 3, 6 or 9 years).

The abundance of nitrosobacteria detected in previously cultivated soil was lower than that observed in fallow soil, with no significant differences observed between 9, 6, and 1 year intervals. The abundance of *Azotobacter* after 9 years of planting was similar to that observed in fallow soil but was much higher than that observed in soil with other planting intervals. The number of aromatic-decomposing bacteria was the highest in fallow soil and was 5.4–14.5-fold higher than that observed in planted soil. The abundance of cellulose-decomposing bacteria was highest in the 1-year planting interval and decreased with longer planting intervals. The numbers of potassium- and phosphate-solubilizing bacteria were greater in fallow soil than in soil with different planting intervals (Fig. 4).

### Enzyme activity after different planting intervals

The variation in soil enzyme activity among planting intervals was different from that observed in unplanted soil (Table 5). The invertase activity in fallow soil and soil with a 1-year planting interval was higher than that detected in soil with other intervals. The invertase activity showed no pattern, as it was highest at an interval of 3 years but lowest at an interval of 6 years. The cellulase activity was highest in fallow soil and the lowest in soil with an interval of 3 years. The catalase activity in fallow soil was higher than that in soil with different planting intervals and decreased with longer planting intervals. The trend in phosphatase activity with respect to pH was acidic >neutral >alkaline. The activity of polyphenol oxidase showed no regular trend, being it was highest at the interval of 3 years and lowest at the interval of 1 year.

**Table 5:** The enzyme activities detected after different interval year.

Interval Year	Invertase (mg/(g·24 h))	Amylase (mg/(g·24 h))	Cellulase (mg/(10 g·72 h))	Catalase (0.1 N·KMnO_4_ ml)	Peroxidase (mg/(g·2 h))
unplanted	137a	5.40b	57.3a	1.89a	3.60c
9 years	114b	6.97a	49.4b	0.547b	10.9a
6 years	112b	3.30c	47.8b	0.865c	10.4a
3 years	108b	7.57a	33.4c	0.929c	7.50b
1 year	125a	4.75b	54.1ab	1.05d	6.67b
**Interval Year**		**Phosphatase (mg/(g·24 h))**			**Polyphenol Oxidase (mg/(g·2 h))**
		**acidic**	**neutral**	**alkaline**	
unplanted		322a	211a	39.3a	1.82b
9 years		299c	85.9c	23.7b	1.43bc
6 years		235d	46.7e	17.0c	1.84b
3 years		209e	67.4d	11.9d	3.36a
1 year		325a	123b	23.7b	1.30c

Notes: Different lowercase letters indicate significant differences (n = 9, *p < *0.05).

## Discussion

The most abundant group of soil microorganisms are bacteria, which play important role in the conversion of soil organic and inorganic matter. Bacteria diversity is affected by soil conditions, seasonal plants, and age^32^. Fungi are some of the primary members involved in the decomposition of soil organic matter and humus, which have an effect on soil fertility. Furthermore, the number and composition of fungi are influenced by the amount of organic present matter in soil^33^. Members of the phylum Actinomycetes produce nitrogen-containing and nitrogen-free organic compounds in soil and can even decompose soil humus. Actinomycetes are closely associated with soil fertility and plant disease control^34^. In the present study, the numbers of bacteria and actinomycetes in soil with *P. notoginseng* were lower than those detected in fallow soil, whereas the numbers of fungi showed no significant difference. Previous studies reported that crop rotation can improve soil microbial structure, which is reflected by an increase by the numbers of bacteria and actinomycetes and a decrease in the numbers of fungi^35^. In the present study, except for the fallow soil, the numbers of actinomycetes increased with longer planting intervals. Except for the 1-year- interval samples, the abundance of fungi showed no significant difference. Planting *P. notoginseng* changed the soil microecology, possibly because *P. notoginseng* releases organic matter into the soil and absorbs inorganic compounds present in soil, which ultimately leads to changes in the structure, ventilation, and biological activity of the soil. Root exudates have inhibitory or promoting effects on microorganisms in the soil. A series of interactions from antagonistic to synergistic occur among the microorganisms and between microbes and plants, resulting in changes in the microbial community structure in the soil. Changes in the community structure of soil microorganisms also results in changes in nitrosobacteria, *Azotobacter*, aromatic-decomposing bacteria, cellulose-decomposing bacteria phosphate- and potassium-solubilizing bacteria.

Soil enzyme activity is also used as an indicator of changes in the quality and productivity of soil^36^ and is used to study the short and long-term effects of a disturbance, as the biochemical reactions in soil are mediated by microorganisms^37^. These reactions are catalyzed by enzymes that play an important role in biogeochemical cycles^38^. The results of the present study showed that the activities of invertase, amylase, catalase, polyphenol oxidase and phosphatase in soil with *P. notoginseng* was lower than those detected in fallow soil, whereas the activities of cellulose, urease and peroxidase did not exhibit a regular pattern. The activities of invertase, cellulase, and catalase in fallow soil were higher than those detected in soil with different planting intervals. However, the activity of peroxidase in fallow soil was lower than that detected in soil with different planting intervals. The decrease in invertase, amylase, and cellulase activities associated with the carbon cycle may result from long-term planting of *P. notoginseng*, which reduces the capacity for soil carbon cycling and the decomposition of specific sugars, starch, and cellulose. A decrease in catalase activity in soil with *P*. notoginseng is not conducive to the decomposition of hydrogen peroxide in the soil, which may lead to toxic effects of hydrogen peroxide on plants. Soil polyphenol oxidases play a role in the decomposition of phenolic acids in soil. With an increasing duration of cultivation, a decrease in polyphenol oxidase activity leads to a decrease in the capacity for phenolic acid decomposition. Phenolic acids are allelochemicals, an increase in which may be one of the reasons for the development of continuous cropping as the number of years of planting increases.

Soil fertility refers to the ability of the soil to support plant growth and coordinate nutrient conditions and environmental conditions, including effective and potential fertility^39^. In the present study, the amounts of organic matter, available N, Ni, Cu, Zn and Fe in soil with *P*. notoginseng were lower than those detected in fallow soil. However, the amount of water-soluble Ca was higher than that observed in fallow soil, whereas the amount of available N, available P, available Mn and water-soluble Mg showed no regular pattern. The results showed that the amount of organic matter in soil with *P. notoginseng* was lower than that present in fallow soil. On the one hand, this pattern may be due to decomposition by microorganisms in the soil, as this process converts soil organic matter into inorganic components that can be used by plants. Alternatively, this pattern may have an anthropogenic cause, as the removal of debris is another reason for reduced organic matter in soil, and this process eventually leads to a decrease in the soil organic matter content.

## Conclusion

The effects of continuous cropping were complex, resulting from the combined effects of three complex systems: plants, soil, and microorganisms. By studying different soils with *P. notoginseng*, we observed that the ecological structure of microbes and the balance of soil nutrients changed. These changes in the soil ecosystem prevented the soil from adapting to the continuous cropping of *P. notoginseng*, which eventually led to the inhibition of *P. notoginseng* growth and increased the incidence of diseases. Soil organic fertilizers, microbial fertilizers, and essential trace elements could be used to supplement the missing elements in the soil, to restore the balance of the soil and alleviate the obstacles associated with continuous cropping of *P. notoginseng*.

## Data Availability

The data used to support the findings of our study are presented in the article and readers can assess it through the article content. The raw data can be requested from the corresponding author via e-mail.
